# Ternary nanocomposites of reduced graphene oxide, polyaniline and hexaniobate: hierarchical architecture and high polaron formation

**DOI:** 10.3762/bjnano.9.272

**Published:** 2018-11-26

**Authors:** Claudio H B Silva, Maria Iliut, Christopher Muryn, Christian Berger, Zachary Coldrick, Vera R L Constantino, Marcia L A Temperini, Aravind Vijayaraghavan

**Affiliations:** 1 Department of Fundamental Chemistry, Institute of Chemistry, University of São Paulo, Av. Prof. Lineu Prestes 748, São Paulo 05508-000, Brazil; 2 School of Materials and National Graphene Institute, The University of Manchester, Booth St E, Manchester M13 9PL, United Kingdom; 3 present address: Department of Physical Chemistry, Institute of Chemistry, Federal University of Bahia, Rua Barão de Jeremoabo, 147, Salvador 40170-115, Brazil; 4 School of Chemistry and Photon Science Institute, The University of Manchester, Alan Turing Building, Oxford Rd, Manchester M13 9PY, United Kingdom; 5 School of Electrical and Electronic Engineering, The University of Manchester, Sackville Street Building, Manchester M1 3BB, United Kingdom

**Keywords:** graphene oxide, hexaniobate, polyaniline, Raman spectroscopy, secondary doping

## Abstract

Nanostructured systems, such as nanocomposites, are potential materials for usage in different fields since synergistic effects of their components at the nanoscale domain may improve physical/chemical properties when compared to individual phases. We report here the preparation and characterisation of a new nanocomposite composed of polyaniline (PANI), reduced graphene oxide (rGO) and hexaniobate (hexNb) nanoscrolls. Atomic force microscopy images show an interesting architecture of rGO flakes coated with PANI and decorated by hexNb. Such features are attributed to the high stability of the rGO flakes prepared at room temperature. Detailed characterisation by X-ray photoelectron and Raman spectroscopies indicates an intermediate reduction degree for the rGO component and high doping degree of the PANI chains compared to the neat polymer. The latter feature can be attributed to cooperative effects of PANI chains with rGO flakes and hexNb nanoscrolls, which promote conformational changes of the polymer backbone (secondary doping). Spectroscopic and electrochemistry data indicate a synergetic effect on the ternary nanocomposite, which is attributed to interactions between the components resulting from the morphological aspects. Therefore, the new nanocomposite presents promising properties for development of new materials in the film form on substrates for sensing or corrosion protection for example.

## Introduction

Nanostructured systems, such as nanocomposites, are potential materials for usage as electrochemical (bio)sensors for analytical purposes, electronics, energy storage devices and corrosion protection because the synergistic effects of their components at the nanoscale range may improve physical/chemical properties when compared to individual phases or enable new technological applications [1–3]. For instance, ternary nanocomposites (conducting polymers, metal oxides and carbon-based materials) exhibit improved energy and power densities, improved stabilities upon charge/discharge cycles [4], and higher electrocatalytic activity in the quantification of chemical species compared to the isolated components [5].

In earlier studies, some of us reported the preparation of binary nanocomposites of polyaniline (PANI) and hexaniobate (hexNb) nanoscrolls by layer-by-layer assembly and the characterisation by spectroscopic and cyclic voltammetry/spectroelectrochemical techniques [6]. The inorganic phase induces a secondary doping of the conductor polymer. In another work [7], Raman and EPR spectra also revealed that a PANI/hexNb nanocomposite prepared by the self-assembly approach exhibits a higher conversion of bipolaronic to polaronic segments compared to the neat polymer and a superior thermal stability (the doped form of PANI is observed even after heating at 150 °C for 90 min).

The literature has shown that PANI and reduced graphene oxide (rGO) show enhanced properties when combined at the nanoscale domain and applied, for instance, as supercapacitors, sensing materials, solar cells, electrochromic devices, anticorrosion coatings or as materials for carbon dioxide capture [8]. The layered inorganic phase offers a high surface area for PANI deposition and increases its thermal stability with regard to decomposition, which is assigned to strong interactions between the two components [8]. Besides, rGO presents defects and functional groups on its surfaces that are sites for catalysis or sorption of substrates [9]. Considering the confirmed synergistic effects of PANI/hexNb and PANI/rGO binary nanocomposites, materials comprising all three components can be expected to show improved properties.

**Figure 1:**
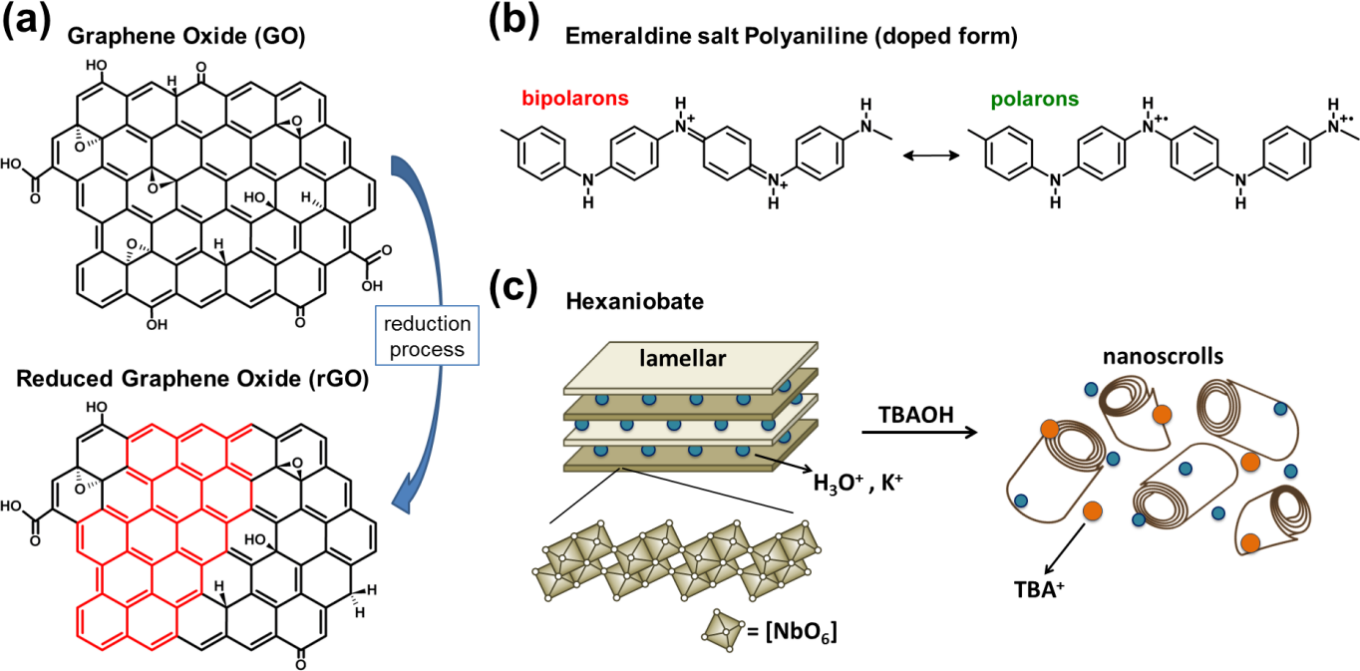
Schematic representation of (a) graphene oxide and reduced graphene oxide structures, (b) polaron and bipolaron segments of emeraldine salt polyaniline (doped form), and (c) hexaniobate in layered and nanoscroll morphologies. TBAOH: tetra(*n*-butyl)ammonium hydroxide, TBA^+^: tetra(*n*-butyl)ammonium cation.

Since its discovery in 2004, graphene has been proposed for a wide range of applications due to its supreme values of specific surface area, electronic mobility, thermal and electrical conductivities and elastic modulus [10–11]. Graphene oxide (GO) is a graphene derivative that has also attracted great scientific interest due to its better processability and scalable production in comparison with pristine graphene [12]. The great chemical versatility of GO is mostly attributed to its complex structure, composed of 2D carbon layers with several oxygen-containing groups, such as hydroxy, epoxy, carbonyl and carboxyl, as schematically shown in Figure 1a [12–13]. Moreover, stable aqueous dispersions containing large GO flakes (above 20 μm) can be prepared [14]. For some applications, the restoration of the hexagonal carbon lattice (removal of functional groups) may be required and this process is performed by thermal or chemical reduction of GO, resulting in reduced graphene oxide (rGO) in which some of the properties of graphene are almost recovered, such as mechanical resistance and thermal and electrical conductivities [15–17].

Polyaniline (PANI) is a conducting polymer that has shown promising properties for the development of materials for different fields such as chemical sensing [18–19], memory devices [20–21] and energy storage [22–23]. As schematically shown in Figure 1b, the conducting form of PANI, the emeraldine salt (PANI-ES) contains two species: unpaired spin segments, the polarons (also known as radical cations); and paired spin segments, the bipolarons (also known as dications) [24–25]. The combination of PANI and inorganic materials at the nanoscale has shown interesting results for the preparation of nanocomposites that exhibit improved electrochemical, mechanical or thermal behaviour [8,26–27].

Hexaniobate (hexNb) is a semiconducting metal oxide composed of negatively charged layers of [NbO_6_] octahedral units and interlayer cations, such as potassium ions (precursor form K_4_Nb_6_O_17_) and protons (acidic form H_2_K_2_Nb_6_O_17_) [28–29]. Due to their high acidic surfaces, protonic niobates and titanoniobates have been reported as promising solid acid catalysts for various chemical reactions [30–31]. Moreover, hexaniobate can be exfoliated by treatment with a number of species such as *n*-alkylamines and tetra(*n*-alkyl)ammonium hydroxides, giving rise to colloidal dispersions of nanoparticles of different morphologies such as nanosheets and nanoscrolls [32–33]. As reported previously [6], the treatment of H_2_K_2_Nb_6_O_17_ with tetra(*n*-butyl)ammonium hydroxide (TBAOH) is an efficient method to produce dispersions of protonic hexNb nanoscrolls (schematised in Figure 1c). Moreover, strong interactions of PANI chains with acidic surfaces of H_2_K_2_Nb_6_O_17_ may dramatically affect the doping state of the polymeric chains [6–7], which is an interesting feature regarding applications of PANI-based materials.

In this paper we report the development of a new nanocomposite, with interesting nanostructured features, composed of reduced graphene oxide, polyaniline and hexaniobate, as well as its spectroscopic and textural characterisation.

## Results and Discussion

GO reduction at high temperatures (typically above 90 °C) results in the aggregation of the material due to restacking of the hydrophobic rGO layers [34–35]. This is a drawback in the preparation of rGO nanocomposites because phase segregation and/or composition heterogeneity will occur in the final materials. Although it is well known that high reaction temperatures are important for the degree of reduction of the resulting rGO, samples prepared at room temperature (25 °C) using longer reaction times (7 days) may present satisfactory properties for nanocomposites. To obtain stable mixture of the components and optimize the interaction between them, graphene oxide reduction was performed at diluted conditions with hydrazine at low temperature (see Experimental section). The resulting dispersions of rGO-25 and rGO/PANI nanocomposites are remarkably stable (see Supporting Information File 1), more so than sample rGO-80. This indicates that the nanocomposites may exhibit low compositional heterogeneity and possibly strong interactions (such as electrostatic and π–π interactions) between their components.

**Figure 2:**
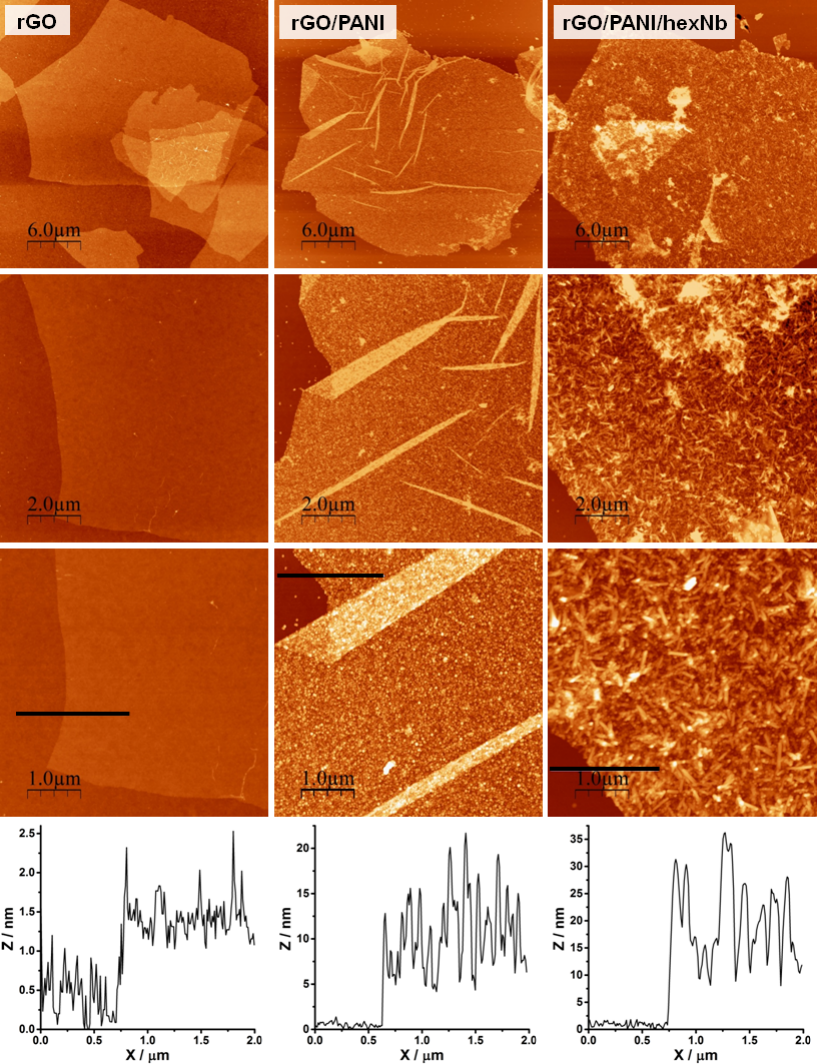
AFM images of rGO-25 sample, and rGO/PANI and rGO/PANI/hexNb nanocomposites at different scan sizes (30, 10 and 5 μm), and height profiles (for the 5 μm scan size images).

The morphological characterisation of rGO-25, rGO/PANI and rGO/PANI/hexNb samples was carried out by atomic force microscopy, as shown in Figure 2. AFM images of the rGO-25 sample show particles of well-defined edges and size ranging from 5 to 25 μm. The height profile (Figure 2, right column) shows thickness of ca. 1.0 nm and a surface roughness (RMS) of 0.24 nm for the rGO flake. These results clearly indicate the presence of smooth monolayer rGO particles, which are partially restacked when deposited on the Si/SiO_2_ substrate. The AFM images of the rGO/PANI nanocomposite show similar flake dimensions (ca. 25 μm) as the rGO-25 sample, and no granular particles were observed, as reported for PANI aggregates [36]. On the other hand, this nanocomposite presents several creases and folds and, more interestingly, shows higher flake thickness and higher surface roughness (ca. 10 and ca. 4.0 nm, respectively). These results clearly indicate that the deposition of PANI on rGO flakes induces an increase of the surface heterogeneity. Analogously, the AFM images of rGO/PANI/hexNb also indicate the presence of large flakes in the nanocomposite and, as shown by the 5 μm scan-size image (and corresponding height profile), the flake thickness and surface roughness are ca. 19 and ca. 7.2 nm, respectively. These results clearly indicate that the surface heterogeneity of the ternary nanocomposite is even higher than that of rGO/PANI, probably due to the presence of hexaniobate nanoparticles (nanoscrolls) on the rGO/PANI flakes.

**Figure 3:**
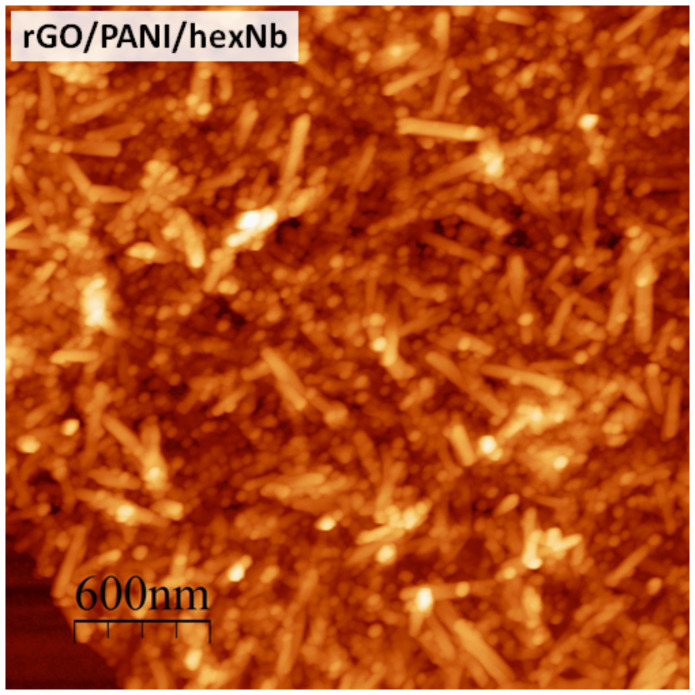
AFM images of rGO/PANI/hexNb nanocomposite at 3 μm scan size.

For further description of the rGO/PANI/hexNb nanocomposite morphology, Figure 3 presents the AFM image of the hybrid material at 3 μm scan size. Figure 3 shows that the particles on the surface of rGO/PANI/hexNb exhibit a scroll-like shape (high aspect ratio), which is very similar to well-described hexNb nanoscrolls [7,37–38]. These results show the interesting hierarchical architecture of the ternary nanocomposite of rGO flakes coated with PANI and decorated by hexNb nanoscrolls.

**Figure 4:**
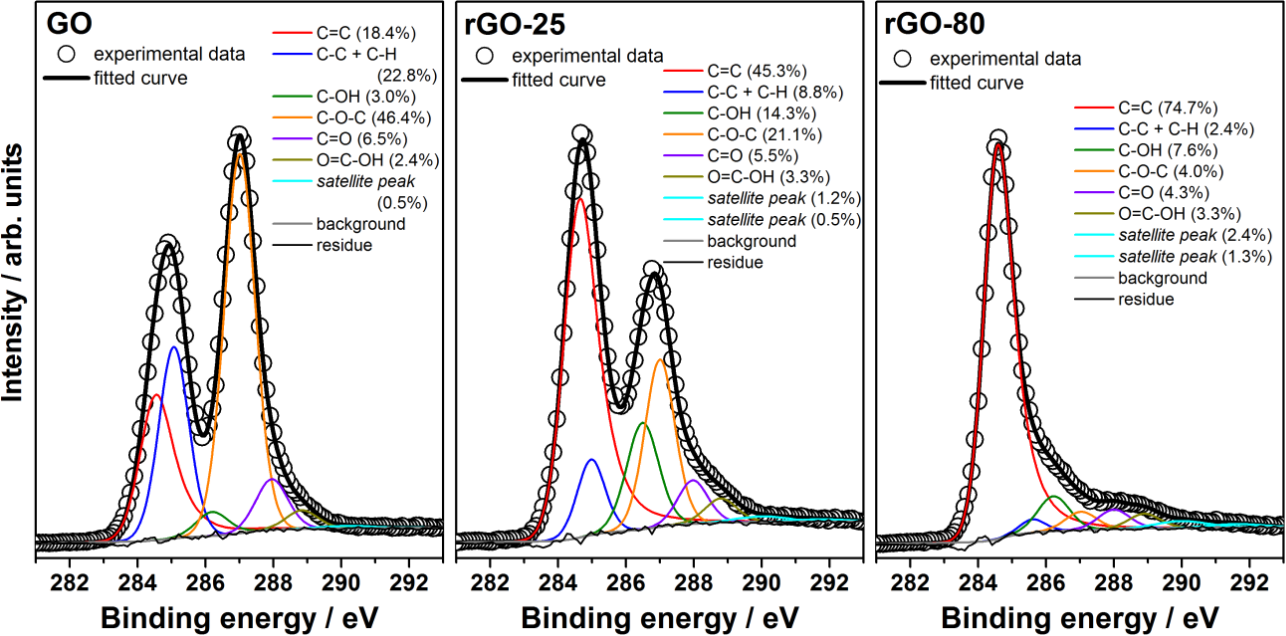
XPS spectra at the C 1s core level of GO and rGO samples prepared by reactions at 25 °C for 7 days or at 80 °C for 3 h (rGO-25 and rGO-80, respectively).

To analyse the reduction of graphene oxide under the present conditions, GO and rGO samples were characterised by XPS spectroscopy. High-resolution XPS spectra can also provide information on the reduction degree of GO, since C 1s core level photoelectrons present slightly different binding energies depending on the environment of the carbon atoms. Figure 4 shows the high-resolution XPS spectra at the C 1s core level for GO and rGO samples prepared by reactions at 25 °C for 7 days and at 80 °C for 3 h (rGO-25 and rGO-80, respectively). XPS spectra of GO and rGO-25 consist mainly of two asymmetric and highly overlapping peaks (maxima at ca. 285 and ca. 287 eV). The comparison of these spectra clearly shows the increase of relative intensity of the low-binding energy peak upon reduction. This is attributed to the partial recovery of the sp^2^-hybridized carbons in the graphene structure, since pristine graphite presents only an asymmetric peak at ca. 284 eV (carbon atoms in sp^2^ environment) [39–41]. The curve fitting of the C 1s spectra, also presented in Figure 4, can provide detailed information on the oxygen-containing groups, since these groups induce different environments for the carbon atoms and, consequently, their corresponding C 1s photoelectrons present slightly different binding energies [34,39–40,42–51]. The comparison of the curve fitting for GO and rGO-25 shows the increase of the contribution from sp^2^ carbons (C=C) and hydroxy groups (C–OH) upon reduction, and the decrease of sp^3^ carbons (C–C + C–H) and epoxy groups (C–O–C). These results are in good agreement with the literature and indicate the recovery of the sp^2^ carbon atoms from the sp^3^ carbon atoms and epoxy groups, and the conversion of some epoxy to hydroxy groups [28,45–46,48]. In contrast, rGO-80 presents a dramatic change in the C 1s spectral profile, evidenced by an intense peak at ca. 284 eV and a weak shoulder at 285–290 eV. The comparison of the curve fitting for this sample and rGO-25 shows a significant increase of the contribution from the sp^2^ carbon atoms and decrease of the contribution from sp^3^ carbon atoms, hydroxy and epoxy groups. These features are very similar to data reported in literature for chemically reduced GO [34,39,42–51] and indicate a high degree of reduction of the rGO-80 sample. This also points out the very important role of the temperature on the recovery of the sp^2^ carbon network in graphene oxide.

**Figure 5:**
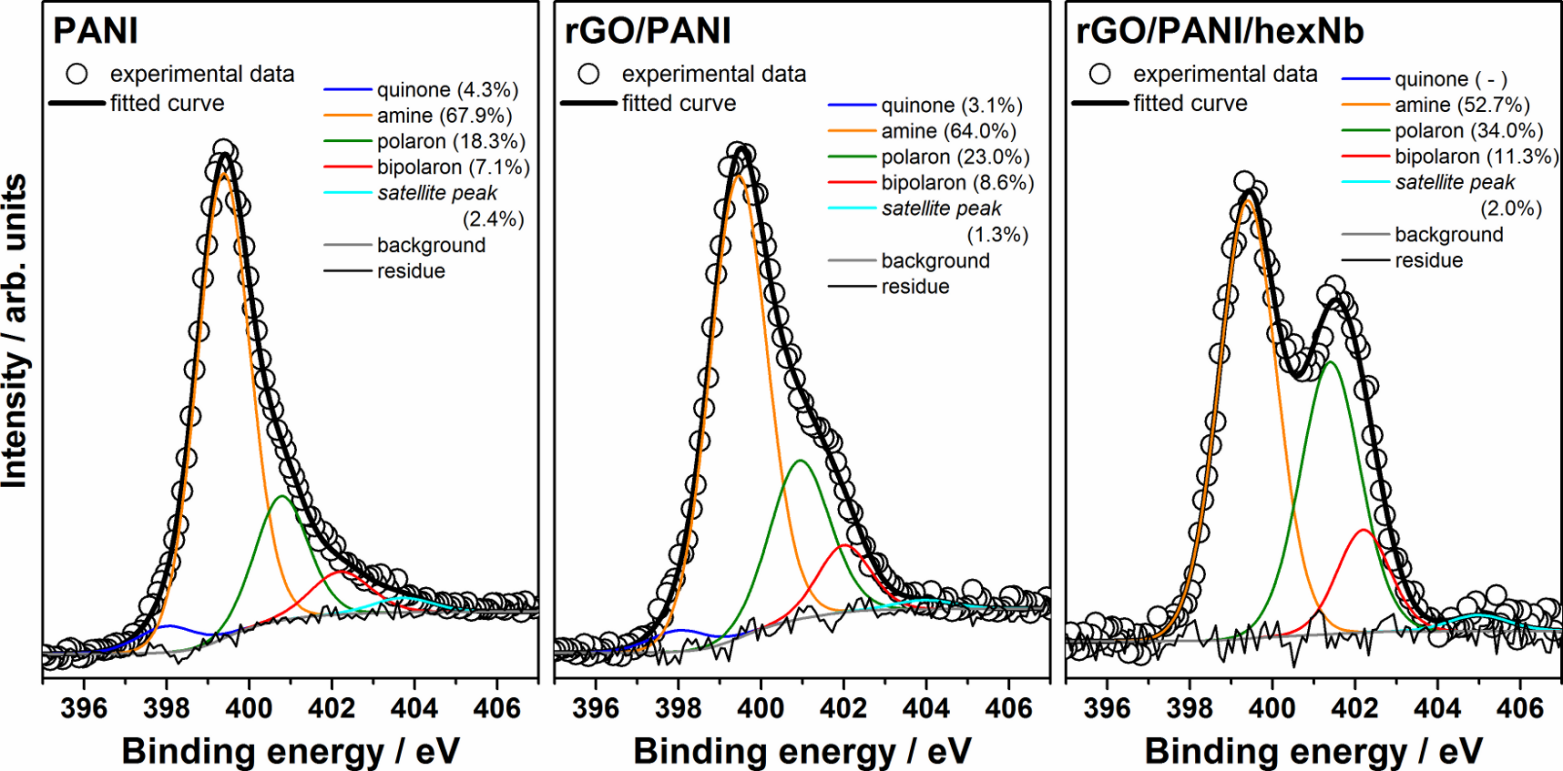
XPS spectra at N 1s core level of PANI and nanocomposites rGO/PANI and rGO/PANI/hexNb.

Analogously to C 1s, XPS spectra at the N 1s core level can be discussed in detail and provide interesting structural information on the rGO/PANI and rGO/PANI/hexNb nanocomposites. In the present study, the N 1s peaks are mostly from nitrogen-containing groups of PANI, the amine, imine or charged nitrogen sites (from polarons or bipolarons) of the polymeric chains [52–55]. Therefore, XPS spectroscopy provides important information on the oxidation and doping states of the polymer in PANI-based materials. Figure 5 shows XPS spectra at the N 1s core level of PANI and rGO/PANI and rGO/PANI/hexNb nanocomposites, and the respective curve fitting results. The N 1s peak of PANI-based materials is dominated by an amine (–NH–)-related component at ca. 399 eV, but also shows components of quinone (=N–), polaron (–N^•+^H–) and bipolaron (=N^+^H–) groups at ca. 398, ca. 401 and ca. 402 eV, respectively. The comparison of spectral data for PANI and rGO/PANI in Figure 5 shows that the relative contributions of polarons and bipolarons are higher in the nanocomposite. These results indicate that PANI chains present a higher doping state in the presence of rGO flakes. XPS data for the rGO/PANI/hexNb nanocomposite clearly shows a significant increase in the relative intensity of the peak with higher biding energy, attributed to polarons and bipolarons. Fitting results show that relative contributions of polarons increase from 18.3% in PANI and 23.0% in rGO/PANI to 34.0% in the ternary nanocomposite. Also, relative contributions of bipolarons increase from 7.1% in PANI and 8.6% in rGO/PANI to 11.3% in the rGO/PANI/hexNb nanocomposite. These results clearly indicate that hexNb nanoparticles also play an important role to the increase of the doping state of PANI. More interestingly, fitting results indicate that the ratio between polarons and bipolarons is higher for the ternary nanocomposite (polaron/bipolaron = 3.0), compared to PANI (2.6) and the binary nanocomposite (2.7). This indicates that the formation of polaron segments in the PANI chains is further induced by the interaction with rGO and hexNb components. This behaviour of high polaron formation induced by hexNb nanoparticles has been reported by us before [7]. The results of XPS spectra at N 1s core level are supported by resonance Raman spectroscopy, as shown below.

**Figure 6:**
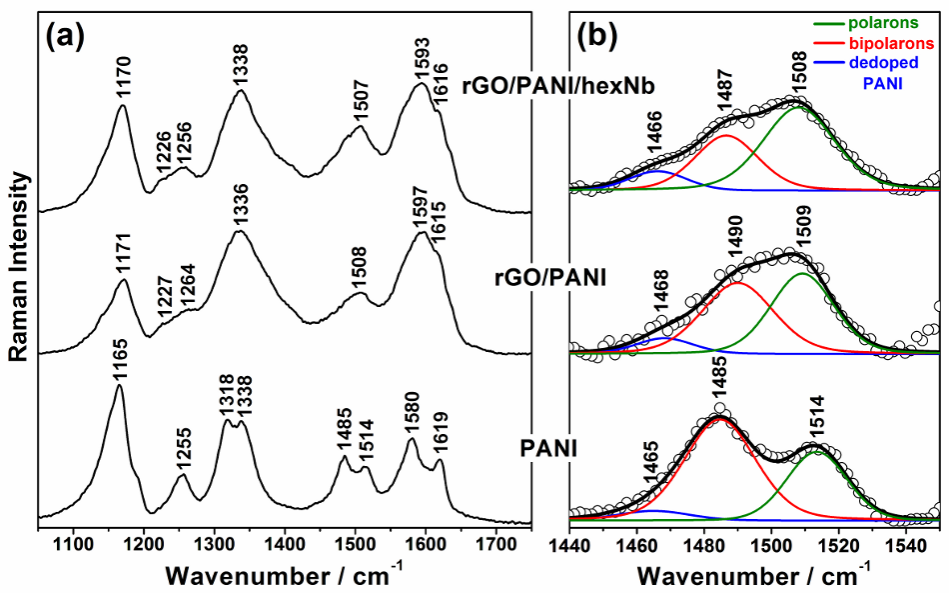
(a) Raman spectra (λ_0_ = 632.8 nm) of PANI, rGO/PANI and rGO/PANI/hexNb in the range of 1050–1750 cm^−1^ and (b) curve fitting for bipolaron and polaron components in the range of 1440–1550 cm^−1^.

The structural characterisation of PANI in the nanocomposites was also performed by Raman spectroscopy, as presented in Figure 6. Raman bands in the spectra of rGO/PANI and rGO/PANI/hexNb at 632.8 nm excitation wavelength are mainly attributed to the polymer due to resonance effects with the polaronic/bipolaronic electronic transitions of PANI, and the high content of polymer in these materials [55–58]. The spectra presented in Figure 6a show the characteristic features of the emeraldine salt form (doped polymer) for all samples. However, comparing the spectra of the nanocomposites with the neat polymer (PANI), the bands at ca. 1336 and ca. 1600 cm^−1^ for the hybrid materials present higher relative intensities. These results can be attributed to the contribution of rGO bands [35–36,45–46,48,50–51,59–60], and changes in the doping state of the polymer due to interaction with the other components.

Figure 6b presents the curve fitting results in the range of 1450–1550 cm^−1^. The component at lower wavenumber (red curve) is assigned to bipolaron segments, whereas the component at higher wavenumber (green curve) is assigned to polaron segments. The comparison of the results for PANI and rGO/PANI shows the increase of the relative intensity of the polaron component, which indicates a conversion of bipolarons to polarons in the presence of the rGO flakes. Moreover, Figure 6b shows that the relative contribution from the polaron segments is further increased for the rGO/PANI/hexNb sample, which suggests a higher formation of polarons induced by hexNb nanoparticles. These results are in agreement with XPS data previously discussed and reports of spectroscopic characterisation of PANI-based materials [6–7,26,61], which suggested a high doping level of PANI induced by conformational changes in the polymeric backbone.

**Figure 7:**
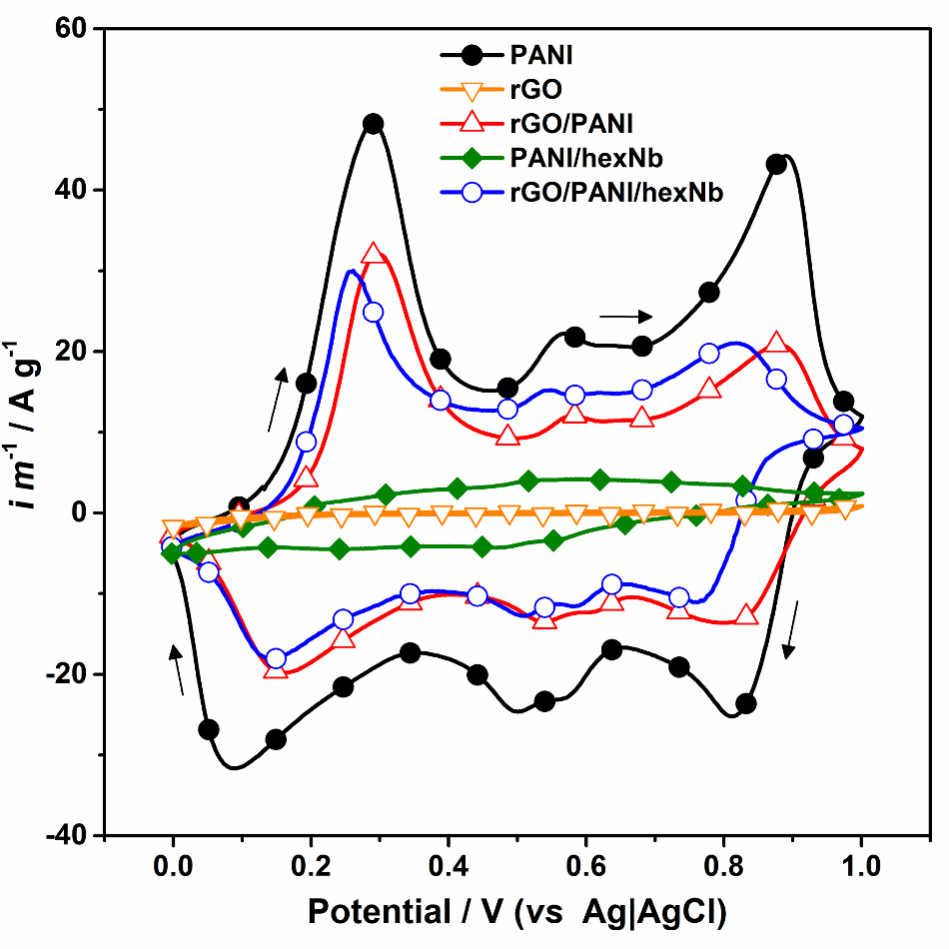
CV curves of PANI, rGO, rGO/PANI , PANI/hexNb and rGO/PANI/hexNb at 25 mV·s^−1^ scan rate. Electrolyte solution: 1 mol·L^−1^ sulfuric acid.

Figure 7 presents the CV curves obtained for PANI, rGO, rGO/PANI, PANI/hexNb and rGO/PANI/hexNb in 1 mol·L^−1^ sulfuric acid solution. For PANI sample and nanocomposites, Figure 7 shows the characteristic redox peaks at ca. +0.30 V (+0.10 V), attributed to the oxidation (reduction) processes between leucoemeraldine and emeraldine salt states, and the peaks at potentials above +0.75 V attributed to the transitions between emeraldine salt and pernigraniline states [62–64]. The peaks at intermediate potentials (between +0.40 and +0.70 V) are attributed to oxidative electrochemical reactions initiated when PANI is cycled at higher potentials than +0.70 V. The CV curve for rGO sample confirms the absence of significant faradaic processes due to a very low current profile. For the PANI/hexNb reference sample, the CV curve presents less defined oxidation (reduction) peaks at +0.20 V (+0.25 V) and +0.80 V (+0.75 V), in addition to intermediate peaks in the range of +0.40–0.60 V. This low current potentiodynamic profile is attributed to the absence of faradaic processes in the hexaniobate component, which also affects polyaniline redox processes under the experimental conditions. The comparison of the curves of rGO/PANI and rGO/PANI/hexNb in Figure 7 indicates similar areas, although neat PANI still presents the highest current profile. The specific capacitances calculated for PANI, binary rGO/PANI and ternary rGO/PANI/hexNb are 880, 515 and 564 F·g^−1^, respectively, whereas for the binary PANI/hexNb reference sample it is 87 F·g^−1^. The difference between the specific capacitances of neat PANI and nanocomposites can be attributed to the presence of rGO. Surprisingly, the comparison of binary and ternary nanocomposites performances indicates that hexNb nanoparticles improve the electrochemical properties of the ternary material under the experimental conditions. Considering that PANI/hexNb sample presents a lower current profile, the observed improvement for rGO/PANI/hexNb indicates a synergetic effect, which is attributed to strong interactions between the components in the ternary nanocomposite. These results are in accordance with structural characterisation presented in this paper, which showed an enhancement of the polymer doping state, due to the secondary doping of the PANI backbone in the rGO/PANI/hexNb nanocomposite. The capacitances of the new nanocomposite presented in this work are comparable to other materials based on graphene oxide/polyaniline reported in literature, which present typical capacitances ranging from 350 to 800 F·g^−1^ [4,22,49,65]. Although this result does not stimulate studies focusing on the application of rGO/PANI/hexNb in energy-storage devices, the thin film obtained by dropcasting has potential to be explored for other purposes since the amount of charge carriers is increased in the ternary nanocomposite.

## Conclusion

In this paper we reported the development a new nanocomposite composed of reduced graphene oxide (rGO), polyaniline (PANI) and hexaniobate (hexNb) prepared by mixing the colloidal dispersions of the components. Morphological characterisation showed an interesting architecture at the nanoscale range of rGO flakes coated with PANI and decorated with hexNb nanoparticles. Such organization was attributed to the good stability of the dispersion, which does not present major aggregation and phase separation. The dispersions of the ternary rGO/PANI/hexNb sample can be deposited on surfaces by spincoating or dropcasting. Structural characterisation by XPS spectroscopy indicated an intermediate reduction degree for the rGO component, and a high doping degree of the PANI chains consistent with secondary doping of the polymer. Electrochemical studies by cyclic voltammetry showed that the capacitance of the ternary nanocomposite is higher compared to the binary composites. Such results are attributed to cooperative effects of PANI chains with rGO flakes and hexNb nanoscrolls promoted by the nanostructured architecture, resulting in a high doping degree of polymeric chains. The interesting chemical versatility and significant interactions between the components are attractive features for applications that require chemically functionalised materials in the film form, such as sensing or corrosion protection.

## Experimental

### Materials

Precursor graphite flakes (NGS Naturgraphit GmbH, 300 μm flake size) were used as received. Aniline (Merck) was distilled under reduced pressure prior to use. Niobium pentoxide (Companhia Brasileira de Metalurgia e Mineração, CBMM) was used as received. All other chemicals (Sigma-Aldrich) were also used as received.

### Preparation of graphene oxide and reduced graphene oxide

Graphene oxide was prepared by graphite oxidation according to a modified Hummers method [66–67]. The viscous gel-like dispersion of graphite oxide obtained after oxidation and purification (centrifugation and washing) was diluted with deionized water and the resulting dispersions were high-shear mixed at 7000 rpm for four times (15 min each). This procedure was used to avoid thermal degradation of the sample. The GO particles prepared by this method present flake sizes ranging from 5 to 30 μm [14,17], which are remarkably larger in comparison to GO reported in literature obtained by sonication (less than 10 μm) [34–35,42–44,67]. Reduced graphene oxide was prepared by chemical reduction of GO in 0.25 mg·mL^−1^ dispersions with hydrazine and ammonia solution at 25 °C for 7 days. The resulting rGO dispersion presents suitable stability for the preparation of the nanocomposites. This sample is labelled as “rGO-25” herein. A reference rGO sample was also prepared by GO reduction under similar conditions as rGO-25, but at 80 °C and 3 h of reaction. This sample is labelled as “rGO-80” herein. Detailed experimental procedures are available in Supporting Information File 1.

### Preparation of polyaniline and hexaniobate precursor dispersions

Dispersions of polyaniline in water/*N*,*N*-dimethylacetamide (DMA) were prepared as described in the literature [6,50,68]. The concentration of PANI, the water/DMA volume ratio and the pH values of the dispersions were adjusted considering the procedure for the preparation of nanocomposites. Hexaniobate (hexNb) was prepared as reported previously [6], which results in colloidal dispersions of scrolled hexaniobate nanoparticles, called hexaniobate nanoscrolls. Detailed experimental procedures are available in Supporting Information File 1.

### Preparation of the binary (rGO/PANI) and ternary (rGO/PANI/hexNb) nanocomposites

For preparation of the rGO/PANI nanocomposite, 35 mL of hydrochloric acid solution (pH 3) was slowly added to 25 mL of PANI solution in DMA (2.8 mg·mL^−1^), and the pH value of the resulting dispersion was carefully adjusted to 3 by adding 1 mol·L^−1^ HCl. Then, 70 mL of rGO-25 dispersion (0.25 mg·mL^−1^, pH 8.7) was slowly added to PANI dispersion along with 1 mol·L^−1^ HCl to maintain the pH value of the PANI/rGO mixture in a range of 2.7–3.0. After addition of rGO dispersion, the pH value was adjusted to 2.6 and the mixture was stirred for 5 days at 20 °C. The total volume of HCl solution used for the preparation of rGO/PANI was 8 mL. The rGO/PANI/hexNb nanocomposite was prepared by slowly adding 40 mL of hexNb dispersion (1.13 mg·mL^−1^, pH 6.8) to 45 mL of rGO/PANI mixture. After mixing hexNb and rGO/PANI dispersions, 1 mol·L^−1^ HCl solution was added to re-adjust the pH value to 2.6, and the rGO/PANI/hexNb mixture was stirred for 2 days at 20 °C. A PANI/hexNb reference sample was prepared with the same PANI/hexNb weight ratio as the ternary nanocomposite. rGO/PANI, rGO/PANI/hexNb and PANI/hexNb samples were further processed by centrifugation/washing cycles (14000 rpm, 20 min and HCl solution pH 2.6) to obtain dispersions of a total concentration of 1.0 mg·mL^−1^.

### Characterization

The AFM images were recorded with a Bruker Dimension FastScan probe microscope, operating in tapping mode, with aluminium-coated Si tips (Bruker). Samples were prepared by spincoating the dispersions of rGO, rGO/PANI and rGO/PANI/hexNb (1.0 mg·mL^−1^ total concentration) on Si/SiO_2_ substrates at 3000 rpm (300 rpm·s^−1^ acceleration, 90 s). In order to properly compare the AFM images, processing was performed with the aid of WSxM software (version 4.0 Beta 7.0) [69]. Height profiles were measured with the aid of WSxM software for the processed images and the surface roughness of the particles will be discussed by means of the root mean square (RMS) values calculated from the height profiles.

XPS spectra were acquired on a SPECS custom-built system composed of a Phobios 150 hemispherical electron analyser with 1D detector. The X-ray source was a microfocus monochromated Al Kα (1486.6 eV) source. Spectra were collected with pass energy of 20 eV, and the combined ultimate resolution is 0.5 eV with X-ray source and 20 eV pass, as measured from Ag 3d. The samples were prepared by dropcasting the dispersions (1.0 mg·mL^−1^ total concentration) on 1 cm^2^ square Si substrates and drying under reduced pressure at room temperature. Data analysis was performed with the aid of CasaXPS software (version 2.3.16 PR 1.6). Peak fitting for high-resolution spectra (C 1s and N 1s core levels) was performed by applying tight constrains for binding energy range, full width at half maximum (FWHM) and shape of components, based on a comprehensive assessment of the literature [13,34,39–40,44–47]. Further details of the fitting parameters are presented in Supporting Information File 1.

Raman spectra were obtained on a Renishaw Raman imaging microscope (inVia) equipped with a Leica microscope and a CCD detector. Spectra were excited at 632.8 nm (RL633 Renishaw Class 3B HeNe laser, 12 mW) and samples were focused with a 50× lens. The laser power was kept below 50 μW to avoid thermal degradation of the samples. For better comparison of the relative intensities, spectral baselines were subtracted.

The electrochemical performance of the samples was evaluated by cyclic voltammetry (CV) at 25 mV·s^−1^ scan rate using a μAutolabIII/FRA2 potentiostat/galvanostat (Metrohm Autolab). The measurements were performed with Ag/AgCl and Pt coil as reference and counter electrodes, respectively, and 1 mol·L^−1^ sulfuric acid as electrolyte solution. The working electrodes were prepared by dropcasting the samples on glass/Cr(5 nm)/Au(60 nm) substrates prepared by thermal evaporation.

## Supporting Information

File 1 Additional experimental data.
