# Supplementary material for: Ternary nanocomposites of reduced graphene oxide, polyaniline and hexaniobate: hierarchical architecture and high polaron formation
Source: Beilstein J Nanotechnol. 2018 Nov 26;9:2936–46. doi: 10.3762/bjnano.9.272 (PMC6278754; doi:10.3762/bjnano.9.272)
Supplement: File 1 — Additional experimental data. [file Beilstein_J_Nanotechnol-09-2936-s001.pdf]

BEILSTEIN JOURNAL OF NANOTECHNOLOGY

Supporting Information

for

Ternary nanocomposites of reduced graphene oxide, polyaniline and hexaniobate: hierarchical architecture and high polaron formation

Claudio H. B. Silva, Maria Iliut, Christopher Muryn, Christian Berger, Zachary Coldrick, Vera R. L. Constantino, Marcia L. A. Temperini and Aravind Vijayaraghavan

Beilstein J. Nanotechnol. **2018,** *9,* 2936–2946. doi:10.3762/bjnano.9.272

Additional experimental data

License and Terms: This is a supporting information file under the terms of the Creative Commons Attribution License (http://creativecommons.org/licenses/by/4.0). Please note that the reuse, redistribution and reproduction in particular requires that the authors and source are credited.
The license is subject to the *Beilstein Journal of Nanotechnology* terms and conditions: (https://www.beilstein-journals.org/bjnano)

Preparation of the graphene oxide and reduced graphene oxide

Graphene oxide was prepared by graphite oxidation according to a modified Hummers method [1,2], followed by exfoliation of graphite oxide to graphene oxide by high-shear mixing. This condition of exfoliation is considered mild comparing with sonication because it does not lead to major cleavage of graphite oxide flakes. Precursor graphite flakes (10.0 g) were slowly added to 340 mL of 95% sulfuric acid (Thermo Fisher) and stirred for 1 h, and then 9.0 g of $NaNO_3$ (Sigma-Aldrich) were added. The resulting suspension was stirred for 3 h in order to dissolve $NaNO_3$ pellets. The suspension was cooled with ice/water bath and 45 g of $KMnO_4$ (Sigma-Aldrich) was very slowly added (over 1.5 h). Ice/water bath was then removed and the mixture was stirred for 7 days. The viscous gel-like dispersion of graphite oxide obtained after purification process by washing/centrifugation with dilute H_2SO_4 and H_2SO_4/H_2O_2 solutions, followed by washing/centrifugation with deionized water for several times, as described by Rourke and co-workers [2]. The resulting dispersion was diluted with deionized water and high-shear mixed at 7000 rpm for four times (15 min each). GO particles prepared by this method present 100% of monolayer content and flake sizes ranging from 5 to 30 μm [3,4], which are remarkably larger in comparison to GO reported in literature obtained by sonication (less than 10 μm) [2,5-10].

Reduced graphene oxide (rGO) samples were prepared under different conditions and evaluated for the preparation of the hybrid materials. Different experimental parameters were explored, such as GO concentration (from 0.12 to 0.25 mg/mL), temperature (25 and 80 °C) and time (from 3 h to 7 days). Such parameters are very important both for the stability of the dispersions and the degree of oxidation/reduction of GO to rGO [5-7,10-12]. For the preparation of the nanocomposites describe in this paper, GO stock dispersion was diluted to 0.25 mg/mL, and 90 mL of such dispersion was collected in a 120 mL flask and kept at 25 °C. Then, 400 μL of ammonia solution (Sigma-Aldrich, 25%) were added and the dispersion was stirred for 20 min. Diluted hydrazine hydrate solution (Sigma-Aldrich, 8%, 273 μL) was

slowly added and the mixture was kept under stirring at 25 °C for 7 days. The pH value of the resulting rGO dispersion was 8.7.

Preparation of dispersion of emeraldine salt polyaniline (PANI-ES)

Dispersions of PANI-ES in water/*N,N*-dimethylacetamide (pH 2.5) was prepared following procedure from literature [13,14]. 1.0 g of dedoped polyaniline (PANI-EB) powder was slowly added to 50 mL of *N,N*-dimethylacetamide (DMA) under stirring. The solution was stirred for 18 h at room temperature and the insoluble particles were removed by filtration. The concentration of PANI-EB/DMA solution was determined by UV–vis absorption spectroscopy considering the absorption coefficient at 630 nm per mole of PANI-EB constitutional tetramer unit (ε_{630} = 2.20 × 10^4 $L{\cdot}cm^{-1}{\cdot}mol^{-1}$, or 60.7 $L{\cdot}cm^{-1}{\cdot}g^{-1}$) [15]. The PANI-EB/DMA solution was diluted by addition of DMA to result in a solution of concentration 12 mg/mL. This solution was further diluted with DMA and acidified in order to prepare the nanocomposites, as described in the paper.

Exfoliation of $H_2K_2Nb_6O_{17}$

The exfoliation of protonic hexaniobate was performed by following procedure described by Shiguihara and co-workers [16,17]. 2.0 mg of $H_2K_2Nb_6O_{17}$ (ca. 1.1 × 10^{-3} mol of H^+-hexaniobate) was suspended in 1 L of tetra(*n*-butyl)ammonium hydroxide solution (TBAOH). The concentration of TBAOH solution was 3.2 × 10^{-3} $mol{\cdot}L^{-1}$, resulting in a suspension of molar ratio TBA^+/H^+-hexaniobate of 0.75 (8.3 × 10^{-4} mol of TBA^+/1.1 × 10^{-3} mol of H^+-hexaniobate). The suspension was stirred for 7 days at room temperature, and then rested for 1 day. Finally, the colloidal dispersion of exfoliated hexaniobate containing nanoscrolls was carefully separated from the deposited solid by using a Pasteur pipette. Hydrochloric acid droplets were added in order to adjust the pH value to 6.8. The concentration of the resulting hexNb nanoscrolls dispersion was 1.1 g/L.

Preparation of PANI/hexNb reference sample

A polyaniline/hexaniobate reference sample was prepared by mixing PANI and hexNb dispersions (concentrations of 2.8 mg/mL and 1.12 mg/mL, respectively). The volumes of the dispersions were such that PANI/ hexNb weight ratio was ca. 0.50 (same value as for the ternary nanocomposite).

Additional results

Figure 1 shows the optical images of the dispersions of rGO prepared by reducing GO with hydrazine at room temperature for 7 days (rGO-25) and the corresponding rGO/PANI nanocomposite dispersion, before and after resting for 5 h. The images in Figure 1 show that rGO-25 and rGO/PANI nanocomposite are stable in dispersion, although reduced graphene oxide samples are well known to present high aggregation in aqueous media.

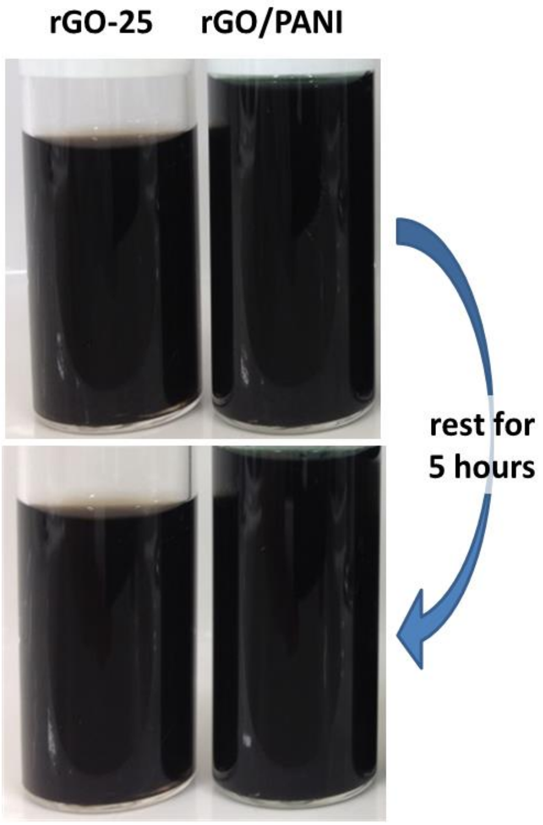

Figure 1: Stability of the rGO and rGO/PANI dispersions before and after resting for 5 h.

As discussed in the paper, XPS spectra at the C 1s and N 1s core levels were fitted in order to provide detailed structural information on the nanocomposite and graphene oxide samples. Peak fitting for such high resolution spectra was performed by applying tight constrains for binding energy range, FWHM and shape of components, based on a comprehensive assessment of the literature [2,7,9,11,12,18-20]. Table 1 and Table 2 show the fitting data and results for the C 1s and N 1s core level spectra, respectively.

Table 1: Fitting data and results of high resolutions XPS spectra at C1s core level of graphene oxide (GO) and reduced graphene oxide (rGO-25 and rGO-80) samples.

Peak assignment	Fitting constrains			GO (RMS = 537.765)			rGO-25 (RMS = 687.175)			rGO-80 (RMS = 755.341)		
	Line shape	Position	FWHM	Position	FWHM	Area %	Position	FWHM	Area %	Position	FWHM	Area %
sp^2 carbons	A(0.30,0.38,20)GL(30)	284.5–284.7	0.9–1.1	284.55	1.10	18.43	284.65	1.10	45.28	284.59	0.96	74.71
sp^3 carbons + C-H	GL(30)	284.9–285.6	0.9–1.1	285.07	1.03	22.83	284.97	0.95	8.84	285.60	0.90	2.39
C-OH	GL(30)	286.2–286.5	0.9–1.1	286.20	1.10	3.04	286.49	1.10	14.31	286.22	1.10	7.65
C-O-C	GL(30)	287.0–287.3	0.9–1.1	287.01	1.06	46.38	287.00	1.03	21.04	287.03	1.10	3.99
C=O	GL(30)	287.6–288.0	0.9–1.1	287.95	1.10	6.44	287.97	1.04	5.51	288.00	1.10	4.30
O=C-OH	GL(30)	288.6–289.0	0.9–1.1	288.84	1.10	2.38	288.80	1.10	3.26	288.89	1.10	3.26
satellite peaks (π-π* shake-up features)	GL(30)	290.0–291.5	1.2–1.8	290.17	1.80	0.51	290.00	1.80	1.24	290.00	1.80	2.45
	GL(30)	291.5–293.0	1.2–1.8	-	-	-	291.71	1.80	0.51	291.61	1.80	1.25

Table 2: Fitting data and results of high resolutions XPS spectra at N1s core level of PANI, rGO/PANI and rGO/PANI/ hexNb samples.

Peak assignment	Fitting constrains			PANI (RMS = 1769.93)			rGO/PANI (RMS = 693.371)			rGO/PANI/ hexNb (RMS = 713.575)		
	Line shape	Position	FWHM	Position	FWHM	Area %	Position	FWHM	Area %	Position	FWHM	Area %
quinone	GL(30)	398.0–398.4	1.5–1.7	398.00	1.70	4.35	398.00	1.70	3.07	398.00	1.70	0.00
amine	GL(30)	399.3–399.8	1.5–1.7	399.37	1.50	67.91	399.45	1.63	64.06	399.40	1.64	52.69
polaron	GL(30)	400.6–401.4	1.5–1.7	400.77	1.51	18.25	400.93	1.70	22.96	401.40	1.70	33.98
bipolaron	GL(30)	402.0–402.7	1.5–1.7	402.20	1.70	7.07	402.00	1.50	8.59	402.20	1.50	11.32
satellite peaks (π-π* shake-up features)	GL(30)	403.0–405.0	1.5–1.7	403.71	1.70	2.42	403.95	1.70	1.32	405.00	1.70	2.01

References

1. Hummers, W. S.; Offeman, R. E. *J. Am. Chem. Soc.* **1958,** *80,* 1339–1339. doi:10.1021/ja01539a017
2. Rourke, J. P.; Pandey, P. A.; Moore, J. J.; Bates, M.; Kinloch, I. A.; Young, R. J.; Wilson, N. R. *Angew. Chem., Int. Ed.* **2011,** *50,* 3173–3177. doi:10.1002/anie.201007520
3. Fiorillo, M.; Verre, A. F.; Iliut, M.; Peiris-Pagés, M.; Ozsvari, B.; Gandara, R.; Cappello, A. R.; Sotgia, F.; Vijayaraghavan, A.; Lisanti, M. P. *Oncotarget* **2015,** *6,* 3553–3562. doi:10.18632/oncotarget.3348
4. Iliut, M.; Silva, C.; Herrick, S.; McGlothlin, M.; Vijayaraghavan, A. *Carbon* **2016,** *106,* 228–232. doi:10.1016/j.carbon.2016.05.032
5. Li, D.; Muller, M. B.; Gilje, S.; Kaner, R. B.; Wallace, G. G. *Nat. Nanotechnol.* **2008,** *3,* 101–105. doi:10.1038/nnano.2007.451
6. Marcano, D. C.; Kosynkin, D. V.; Berlin, J. M.; Sinitskii, A.; Sun, Z.; Slesarev, A.; Alemany, L. B.; Lu, W.; Tour, J. M. *ACS Nano* **2010,** *4,* 4806–4814. doi:10.1021/nn1006368
7. Stankovich, S.; Dikin, D. A.; Piner, R. D.; Kohlhaas, K. A.; Kleinhammes, A. Y.; Wu, J. Y.; Nguyen, S. T.; Ruoff, R. S. *Carbon* **2007,** *45,* 1558–1565. doi:10.1016/j.carbon.2007.02.034
8. Park, S.; An, J.; Piner, R. D.; Jung, I.; Yang, D.; Velamakanni, A.; Nguyen, S. T.; Ruoff, R. S. *Chem. Mater.* **2008,** *20,* 6592–6594. doi:10.1021/cm801932u
9. Rani, J. R.; Lim, J.; Oh, J.; Kim, J.-W.; Shin, H. S.; Kim, J. H.; Lee, S.; Jun, S. C. *J. Phys. Chem. C* **2012,** *116,* 19010–19017. doi:10.1021/jp3050302

10. Fernández-Merino, M. J.; Guardia, L.; Paredes, J. I.; Villar-Rodil, S.; Solís-Fernández, P.; Martínez-Alonso, A.; Tascón, J. M. D. *J. Phys. Chem. C* **2010,** *114,* 6426–6432. doi:10.1021/jp100603h
11. Koinuma, M.; Tateishi, H.; Hatakeyama, K.; Miyamoto, S.; Ogata, C.; Funatsu, A.; Taniguchi, T.; Matsumoto, Y. *Chem. Lett.* **2013,** *42,* 924–926. doi:10.1246/cl.130152
12. Ren, P.-G.; Yan, D.-X.; Ji, X.; Chen, T.; Li, Z.-M. *Nanotechnology* **2011,** *22,* 055705. doi:10.1088/0957-4484/22/5/055705
13. Cheung, J. H.; Stockton, W. B.; Rubner, M. F. *Macromolecules* **1997,** *30,* 2712–2716. doi:10.1021/ma970047d
14. Izumi, C. M. S.; Constantino, V. R. L.; Temperini, M. L. A. *J. Nanosci. Nanotechnol.* **2008,** *8,* 1782–1789. doi:10.1166/jnn.2008.014
15. Moon, D. K.; Ezuka, M.; Maruyama, T.; Osakada, K.; Yamamoto, T. *Macromolecules* **1993,** *26,* 364–369. doi:10.1021/ma00054a016
16. Shiguihara, A. L.; Bizeto, M. A.; Constantino, V. R. L. *Colloids Surf., A* **2007,** *295,* 123–129. doi:10.1016/j.colsurfa.2006.08.040
17. Shiguihara, A. L.; Bizeto, M. A.; Constantino, V. R. L. *J. Braz. Chem. Soc.* **2010,** *21,* 1366–1376. doi:10.1590/S0103-50532010000700024
18. Hsiao, M.-C.; Liao, S.-H.; Yen, M.-Y.; Teng, C.-C.; Lee, S.-H.; Pu, N.-W.; Wang, C.-A.; Sung, Y.; Ger, M.-D.; Ma, C.-C. M.; Hsiao, M.-H. *J. Mater. Chem.* **2010,** *20,* 8496–8505. doi:10.1039/c0jm01679a
19. Bagri, A.; Mattevi, C.; Acik, M.; Chabal, Y. J.; Chhowalla, M.; Shenoy, V. B. *Nat. Chem.* **2010,** *2,* 581–587. doi:10.1038/nchem.686
20. Jeong, H.-K.; Lee, Y. P.; Lahaye, R. J. W. E.; Park, M.-H.; An, K. H.; Kim, I. J.; Yang, C.-W.; Park, C. Y.; Ruoff, R. S.; Lee, Y. H. *J. Am. Chem. Soc.* **2008,** *130,* 1362–1366. doi:10.1021/ja076473o
